# A Systematic Review of the Liaison Nurse Role on Patient’s Outcomes after Intensive Care Unit Discharge

**Published:** 2014-10

**Authors:** Zeinab Tabanejad, Marzieh Pazokian, Abbas Ebadi

**Affiliations:** Behavioral Sciences Research Center, School of Nursing, Baqiyatallah University of Medical Sciences, Tehran, Iran

**Keywords:** Intensive Care Unit, Liaison Nurse, Outcome, Patient, Systematic Review

## Abstract

**Background: **This review focuses on the impact of liaison nurse in nursing care of patient after ICU discharge on patient’s outcomes, compared with patients that are not taken care of by liaison nurses. The role of the ICU liaison nurse has transpired to solve the gap between intensive care unit and wards. Therefore, we aimed to review the outcomes of all studies in this field.

**Methods:** A systematic review of intervention studies between 2004 and 2013 was undertaken using standard and sensitive keywords such as liaison nurse, intensive care unit, and patient outcomes in the following databases: Science direct, PubMed, Scopus, Ovid, Oxford, Wiley, Scholar, and Mosby. Then, the articles which had the inclusion criteria after quality control were selected for a systematic review.

**Results:** From 662 retrieved articles, six articles were analyzed in a case study and four articles showed a statistically significant effect of the liaison nurse on the patient’s outcomes such as reducing delays in patient discharge, effective discharge planning, improvement in survival for patients at the risk for readmission.

**Conclusion:** Liaison nurses have a positive role on the outcomes of patients who are discharged from the ICU and more research should be done to examine the exact function of liaison nurses and other factors that influence outcomes in patients discharged from ICU.

## Introduction

Phrases such as “critical care without walls”, “shifting boundaries”, “bridging the gap” describe the development and improvement of critical care nursing for patients who are in critical and acute health conditions in the hospital and outside the intensive care unit (ICU).^1^ On the other hand, an essential component of most large hospitals is the ICU that is often under pressure because of limited beds.^2^ Patients who are recently discharged from the ICU and transferred to the medical-surgical wards are vulnerable and often need more complex care than other patients in these wards. They are vulnerable to recurrence of acute conditions. In such cases, they may be readmitted to the ICU, which will cause increased inpatient length of stay (at least two-fold increase, 35-47 days),^3^ increased mortality ranging from 1.5 to 10 times, increased treatment and hospital costs (about 15 billion dollars), with a 30% increase of nosocomial infections and development of extra distress for the patients and their families. Research has shown that up to a third of such patients will experience an adverse event after ICU discharge. Half or more of the adverse events occurring after ICU discharge could be preventable with better standards of care.^4^ Furthermore, the ward’s staff might not have enough knowledge and skills to care for these patients properly. In fact, the management of patients recently discharged from the ICU in the medical-surgical wards affects the patients’ outcomes and suitable nursing care is essential for preventing ICU readmission.^5^ Structured and appreciated rehabilitation plans for such patients might start up before discharge from ICU and continue in hospital wards.^4^ For more precise planning for this group of patients, it is better to determine the risk factors for each patient individually according to the preliminary diagnosis and identify the probability of the occurrence of complications, the ICU readmission, common causes of these probabilities and then to arrange the nursing care according to these information.^6^ To solve these problems and also to enhance the efficiency of the role of the ICU nurses for patients recently discharged from the ICU, some hospitals introduced a new position called “liaison nurse” (Australia, 2001) in the hospital.^7^ The liaison nurse is a critical care nurse specialist with knowledge, and skills in providing nursing care to patients in the ICU and in ongoing nursing care to patients in medical-surgical wards.^8^ Their primary purpose is to enable a handicraft base that assists the improvement of critical care specialist practice and elevates high quality nursing care of critically ill patients and their families.^9^ Other advantages of this service include reducing the patient and his family’s anxiety and continuing relationships between the ICU and the medical-surgical wards staff.^1^ Liaison nurse decreases the occurrence rate of adverse changes in patient health conditions and reduces the ICU readmission rate from 2.3% to 0.5%.^10^ In fact, the liaison nurse focuses on improving patients outcomes whether patients are in the ICU or discharged from the ICU and transferred to medical-surgical wards. In addition they provide appropriate nursing care for this group of patients in the medical-surgical wards.^3^One of the crucial and basic roles of the liaison nurse related to patient’s outcomes is to predict the support of patient’s feelings in the transfer phases from the ICU to the medical-surgical wards. The aim is to minimize changes in physical and psychological symptoms in response to the anxiety related to transfer from ICU to the medical-surgical wards^3^^,^^11^ and reduce or prevent ICU readmission.^5^ When the patient is transferred to the medical-surgical wards, the role of liaison nurse on the patient outcomes becomes more concrete and includes training staff to provide suitable nursing care for this group of patients in the medical-surgical wards and to support them in implementing nursing care.^12^ Clinical assessment is the basis for designing proper nursing care for patients in the medical-surgical wards, training the patients and their families as well as supporting them.^13^ The purpose of their assessments is to improve the outcomes of patient care and to prevent ICU readmission.^7^ The aim of this study is to assess the studies on the impact of ICU liaison nurses comprehensively in the outcomes of patients that were discharged from the ICU and finally do a more precise assessment of the differences between the findings of studies. The following research question was addressed: How effective is a liaison nurse as a method of solving the mismatch of nursing care between intensive care unit and wards? ,

## Materials and Methods

*Design*

A systematic review was undertaken using guidelines for identification of quantitative data.^14^We set clear objectives, selection criteria and defined a search strategy for identifying papers. Then, we analyzed the selected studies and synthesized the results of randomized controlled trials.

*Search Methods*

We did a systematic review of studies on liaison nurse impact on the outcomes of patients discharged from the ICU between 2004 and 2013 that had been conducted according to the available resources and documents in English language. For finding published studies in this field, we reviewed journals, conferences, theses, seminars and articles published in foreign journals in databases of Scopus, PubMed, Science direct, Ovid, Oxford, Wiley, Scholar, and Mosby. The systematic search was done using keywords including ‘liaison nurse’, ‘patient’, ‘outcomes’ and ‘intensive care unit’ with other possible combinations of words such as important, original and sensitive. Moreover, to find more relevant articles, the bibliography of the above identified studies were studied. At first, a list of titles and abstracts of all articles in the above mentioned database was prepared. The relevant articles were entered in the research cycle individually. The inclusion criteria included various articles which were in line with the current research, and addressed the liaison nurse impacts on the outcomes of patients discharged from the ICU such as: ICU readmission, length of ICU and hospital stay and patient satisfaction. The exclusion criteria included various articles which addressed other roles of the liaison nurse such as psychological consultation, alcohol liaison nurse and all other articles that did not address liaison nurse impact on patients in the ICU and also were qualitative and descriptive. 

*Search Outcomes*

**Figure 1:**
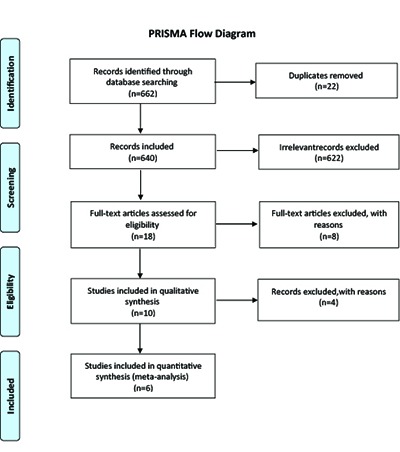
PRISMA Flow Diagram

In total, 662 articles about the liaison nurse’s word in tittle were selected and organized in Endnote Software. Then, 22 article duplicates removed and 640 articles were saved. After reviewing the titles and abstracts from 640 articles, 622 records were excluded because of lack of proper processing of papers and irrelevant to roles of the ICU liaison nurse and 18 records about roles of the liaison nurse in the intensive care unit screened. From 18 relevant records that were identified, 8 records were excluded after assessing the full-text because of poor quality of the content. Finally, 10 records were included in qualitative synthesis. After a careful statistical assessment of the articles, 4 articles were removed because of qualitative statistical methods and six articles of quantitative and interventional records based on inclusion criteria were selected and were included in the final systematic review (figure 1).

*Quality Appraisal*

The studies represented clinical trial using liaison nurse as the transition suitable nursing care from ICU to wards. Two nursing professors analyzed the quality criteria suggested for assessing randomized controlled trials by the Critical Appraisal Skills Programme (CASP) of the Public Health Resource Unit, England, by making sense of the evidence; 10 questions to help make sense of Randomised Controlled Trials (2006).^15^ The quality indicators used to appraise each study are given in table 1. Six studies met the inclusion criteria and were included in the review.

**Table1:** Quality indicators of included studies

Study&origin	Design	sample size	Age group	Focused research question	selection/ allocation	Power calculation/Analysis	Baseline comparability groups	Confounding Factors Considered
Chaboyer et al. (2006) Australia	Comparative Prospective block Intervention	101/85	Adult	Yes	Random group allocation (188 patient)	Too small size. Analysis of Mann-Whitney, mean & standard deviation	Yes	Yes: wash-out period
Chaboyer et al. (2007) Australia	Before-after, Block intervention	62/53	Adult	Yes	Random group allocation (115 patients& 100 families)	No power calculation given, although small sample recognized, Mann-Whitney, test λ2	Yes	Yes: wash-out period
Caffin et al. (2007) Australia	Comparative clinical trial	All patient admitted during July 2004 & June 2005	Children & youth	Yes	Random group allocation (1197 patients)	Power adequate, test λ2	Yes	No such as delayed ICU discharge
S. J. Elliott et al. (2008) Australia	Before-after clinical trial	All patients admitted during 18 months before & after the intervention	Adults	Not given	Random group allocation (835 patient)	Power calculation not given, Mann-Whitney, mean& standard deviation	Not given	No difference in confidence results
Endacott et al. (2010) Australia	Comparative clinical trial	201/187	Adults	Yes	Random group allocation (388patient)	Power adequate, t-test, test λ2	Yes	Yes
T. A. Williams et al. (2010) Australia	Before-after clinical trial	All patients admitted during 6 months before & after the intervention	Adults	Yes	Random group allocation (1435patient)	Power adequate, test λ2	Yes	Yes

*Data Abstraction*

A table was prepared using the criteria established for inclusion in the study (table 2). Key points considered in the review were results descriptors, the design of studies, the validity of measures of assessment, and the duration of assessment.

**Table 2:** Results and evidence of effect of liaison Nurse in nursing care

Study	Clinical Theme	liaison Nurse Intervention	Comparator	Assessment measures	Effect on patients outcomes	Effect on nurses of ICU/wards
Chaboyer et al. (2006)	Discharge Delay after Prolonged ICU Stay	8 h per day, Monday to Friday	Care of liaison nurse	clinical database developed by the Australian & New Zealand Intensive Care Society	Positive impact on the patient planning discharge	This aspect was not investigated
Chaboyer et al. (2007)	Patients and family’s anxiety	8 h per day, up to 4h on the weekend	Care of liaison nurse	STAI scores, anxiety scores	Without statistically significant effect	This aspect was not investigated
Caffin et al. (2007)	Tertiary Pediatric ICU	10 h per day, seven days a week	Care of liaison nurse	Likert scale	Positive effect on relationship, training & Readmission reduction	Positive change to PICU& ward transfers
S. J. Elliott et al. (2008)	Patient outcomes	8 h per day	Impact of an ICU liaison nurse	Australian & New Zealand Intensive Care society, Adult Patient Database experienced, Trained data manager& quality checks	Positive effect on increased throughput & reduction in inpatient days	This aspect was not investigated
Endacott et al. (2010)	Major adverse events	10 h per day, seven days a week	Impact of an ICU liaison nurse	Training to ICU liaison nurse, a chart audit by a Research Assistant	Positive effect on surgical complications Prevention	This aspect was not investigated
T. A. Williams et al. (2010)	Facilitating Discharge from ICU	10 h per day, seven days a week	Effectiveness of a critical care nursing Outreach service	Training to ICU liaison nurse	Without statistically significant effect	This aspect was not investigated

*Synthesis*

Key to the review were result descriptors, the design of studies and the validity of measures of assessment, and the duration of assessment. The key data required were extracted from the articles and recorded in the data extraction form. The data extraction form included: general information related to the article (title and years of studies), characteristics of study (clinical themes, methodology and type of study), and study results. The findings are therefore summarized in a narrative manner rather than using direct comparison. 

## Results

*Characteristics of Studies*

We described the properties and methodology of the systematic studies and then the analysis findings in terms of the liaison nursing impact on the patients outcomes discharged from the ICU will be analyzed. The studies were published between 2004 and 2013 in several journals (Chaboyer et al. 2006; Chaboyer et al. 2007; Caffin et al. 2007; S. J. Elliott et al. 2008; Endacott et al. 2010; T. A. Williams et al. 2010). The year of publication of six analyzed studies shows that we are dealing with a relatively new subject in the field of nursing research, which is liaison nursing (table 1). In the six analyzed studies, 3421 samples were investigated. There were two blind interventional studies (Chaboyer et al. 2006; Chaboyer et al. 2007), three before and after clinical trial studies (Doric et al., 2008, Williams et al., 2010b) and three comparative clinical trials studies (Caffin et al., 2007, Endacott et al., 2010, Chaboyer et al., 2006). Here, we answered two questions: 1- the impact of liaison nurse on the outcomes of ICU patients and 2- whether this impact is statistically significant.

*Liaison Nurse Interventions*

In all the studies, the studied group received the liaison nursing intervention and the control group did not receive any liaison nurse intervention. The shortest and longest liaison nurse intervention was eight and 10 hours per day, respectively, often in every day of the week. The liaison nurse intervention in studies included the patient’s clinical condition assessment, the patients’ and their families’ emotional support, reducing the patients’ and their families’ anxiety, planning and making arrangement for patients discharge from the ICU, ongoing relationship between the medical-surgical wards and ICU, critical care transfer to the medical-surgical wards, and training and clinical support of the ward staff. 

*Evidence of Effectiveness*

The study of Chaboyer et al. in 2006 showed that for patients who received care by a liaison nurse, there was nearly three times less probability of having two-hour delay in discharge from the ICU and there was nearly 2.5 times less probability of having four hours or more delay in discharge from the ICU.^16^ The results of study by Chaboyer et al. in 2007 showed no significant effect of the ICU liaison nurse on the patients’ and families’ anxiety prior to transfer to the wards between the intervention and control groups (P=0.28). However, they suggested that nurses could be helpful in identifying people who were at high risk for anxiety, and asking about the patient’s and family’s feeling about being transferred from ICU could help them.^17^ The study by Caffin et al. in 2007 showed that the readmission rate reduced from 5.4% before liaison nurse intervention to 4.8% after liaison nurse intervention. The very positive results were observed on the role of liaison nurse. 98.5% of the nurses believed that this role was useful and had a valuable impact on the child’s transfer between pediatric ICU and general pediatric ward and 99.5% of parents were satisfied with the role.^3^ The study by S. J. Elliott et al. in 2008 revealed that after the ICU liaison nurse care, self-care abilities of patients increased by 13%. In general, the introduction of ICU liaison nurse led to efficiency of ICU discharge (decreased ICU stepdown days and ICU readmission length of stay) and improved survival for patients who are at risk of readmission.^7^ The study of Endacott et al. in 2010 indicated that the liaison nurse had an effective role in preventing occurrence of complications such as need for surgery (P=0.006) and transfer to the higher level of care (P=0.028).^5^ The study of T. A. Williams et al. in 2010 showed that the liaison nurse role had no impact on the number of hospitalization days in ICU to hospital discharge, ICU readmission and mortality rates, although the studies in this field in Australia and the UK have reported a statistically significant effects of the role of a liaison nurse on patient discharge from the ICU.^18^ Four out of six analyzed studies indicated that the liaison nurse had a statistically significant effect on improvement of outcomes of ICU patient and two studies showed that liaison nurse had no such effect. Considering these contradictions, the question arises whether the liaison nurse is efficient on the outcomes of ICU patient or not?

## Discussion

There have been different studies conducted on the liaison nurse impact in ICU outcomes and each of the studies address a different outcome and the endpoints are different.^5^^,^^7^^,^^18^ Therefore, there is no single conclusion about the liaison nurse impact on one type of outcome in any of the studies; although in all studies, the liaison nurse role was considered as instrumental in improving the continuity of excellent nursing care.^3^^,^^5^^,^^7^^,^^16^^-^^18^ There were many limitations in the studies that were reviewed such as restriction of the hours to evaluate the time delay in discharge from the ICU,^16^ implementation of outcomes in pediatric cardiology wards,^3^ and lack of presence of liaison nursing care on the night shift and weekends.^5^ Exclusion of illiterate parents in pediatric wards was another confounding factor.^3^ Study of Chaboyer et al. and Williams et al. may not have well illustrated the nursing liaison impact on the patient’s and family’s level of anxiety, the number of inpatient days in ICU and hospital, ICU readmission, and mortality rate.^17^^,^^18^ We think that this may have been due to the small sample size of the study. These shortcomings may have been circumvented by conducting their research on a larger sample size. Also Elliot et al. said that ICU liaison nurse roles were more prevalent in larger hospitals with several ICUs and subsequently higher hospitalization and ICU admission rates.^13^ The ICU liaison roles in larger hospitals with high ICU admission rates may function differently with respect to services in smaller hospitals because of the acuity and volume of patients passing through the ICU.^19^ Also among other reasons, it can be mentioned that in order to be effective in reducing the patient’s and family’s level of anxiety, the liaison nurses must be able to detect symptoms and causes of anxiety and provide an appropriate intervention.^17^ Furthermore, counseling skills are also important and liaison nurses in this study with over 20 years of experience in ICU stated that they were not sufficiently prepared to cope with the stressful situations for the patients and their families and they were not instructed on how to communicate with family members.^17^ There are other advantages of liaison nurse services which have been addressed in these analyzed studies. These include: a) increased satisfaction of the patients and their families with the ICU and floor nursing staff, b) improved satisfaction of the ICU and other nurses with the nursing quality of care, c) increased confidence in providing appropriate nursing care for the nurses of both wards, d) quality improvement in discharge planning, e) reduced ICU readmission rates, f) reduced hospital inpatient days, g) increased patient self-care activities, h) accurate assessment from the patient in medical-surgical wards, i) preventing the development of further acute and critical conditions for the patient in medical-surgical wards, and j) the availability of knowledge resources for the wards staff, patient and his family.^5^^,^^13^^,^^18^ Another limitation of the above conclusions was that all of these studies were conducted in Australia and were not implemented in hospitals in other countries.^3^^,^^5^^,^^7^^,^^15^^-^^17^ In general, the results of our systematic review demonstrated that specific and identical statistical conclusions were not introduced for the impact of the liaison nurse on the outcomes of ICU patients such that two of the reviewed studies was not statistically obtained significant effect for nursing care results of the liaison nurse.^17^^,^^18^ Due to the lack of studies in this area, our study attempted to address specifically the liaison nurse impact on the outcomes of ICU patients.

Given the lack of such studies in this field in Iran, small number of clinical trials in this area, the fact that such studies have been exclusively conducted in Australia and a variety of methods to determine the studies impacts on the liaison nurse role, future studies in this field are needed. Also, more comprehensive studies are needed to address other broader issues in which the impact of the liaison nurse on the outcomes of ICU patients, on patient morbidity, and preventing ICU readmissions are addressed. 

## Conclusion

The findings of this systematic study indicated that the different roles of the liaison nurse on the outcomes of patients discharged from ICU are very important especially when compared with patients who do not receive liaison nurse services. However, given the high clinical heterogeneity between studies, the general conclusions should be looked at more carefully.

For this reason, it is suggested that more research be conducted to find the best evidence for the liaison nurse impact and different impacts of these nurses on the outcomes of ICU patients care. Furthermore, more research is needed to be performed in Iran because the concept of the liaison nursing is alien to the nursing profession and needs extensive work in order to familiarize the medical and nursing profession with this concept. 
